# Fabrication of a low-cost benchtop optical imager for quantum dot microarray-based stress biomarker detection

**DOI:** 10.1364/BOE.527338

**Published:** 2024-06-07

**Authors:** Anusha Kishore, Arun Mathew Varughese, Bernhard Roth, Carsten Zeilinger

**Affiliations:** 1 Leibniz University Hannover , Centre of Biomolecular Drug Research, Schneiderberg 38, 30167 Hannover, Germany; 2 Leibniz University Hannover, Hannover Centre for Optical Technologies, Nienburger Str. 17, 30167 Hannover, Germany; 3 Leibniz University Hannover, Cluster of Excellence PhoenixD, Welfenplatz 1, 30167 Hannover, Germany

## Abstract

We report on a simplified optical imager to detect the presence of a stress biomarker protein, namely the Heat shock protein 90 (Hsp90). The imager consists of two elements the optical unit and the sensor, which is a custom-made biochip. Measurement is based on the masking of the streptavidin conjugated quantum dot’s (Sav-QDs) fluorescence when Hsp90 attaches to it via biotinylated antibodies (Ab). The masking effect was directly proportional to the Hsp90 concentration. The cost-efficient benchtop imager developed comprises a CMOS sensor, standard optical lenses, and a narrow bandpass filter for optically eliminating background fluorescence. This approach is promising for the realization of cheap, robust, and reliable point-of-care detection systems for various biomarker analyses.

## 1. Introduction

Hsp90 is an indispensable stress-marker of eukaryotic cells which promotes the survival of the cell under physiological stressful conditions. About 1-2% of the total mammalian cell protein content is Hsp90 under non-stress conditions [1]. It is required to mature and activate many important proteins in the cell [2]. Many diseases induce stress on cellular function while various important cancer proteins are Hsp90 clients [3]. High expression of Hsp90 is observed in several cancer types. Due to preferential secretion by certain types of cancer cells, e.g., extracellular Hsp90 (eHsp90) in blood plasma, it has a high potential for being an early-stage cancer biomarker [2]. Furthermore, Hsp90 plays important roles in the physiology of skeletal muscles, cellular adaptation to exercise or stress, and induction of inflammation [4]. For example, eHsp90, is secreted outside the cell during stressful conditions like heat, hypoxia, harmful irradiation, and tissue injury. It is also involved in mobility and wound healing [2].

Currently, there are few non-invasive tests available for clinical detection of biomarkers. Especially, portable diagnostics can be used for rapid and cheap health monitoring in areas where there is a lack of laboratories or skilled technicians [5]. Various techniques can be used for this purpose, e.g., based on interferometry or plasmonic and Raman fingerprint spectroscopy, to name just a few [6–11]. There is a steadily increasing demand for portable biosensors, hence also an immediate need for biosensor miniaturization [12]. Point-of-care biosensors can be divided into hand-held devices and benchtop devices, which are reduced forms of large laboratory equipment in terms of size and complexity [13–16]. Although ELISA is the gold standard for clinical diagnosis tests for many viral and bacterial diseases some problems with ELISA are the requirements of expensive instruments and skilled professionals as well as long waiting times to obtain the results. Portable diagnostics can be used for rapid and cheap health monitoring in areas where there is a lack of laboratories or skilled technicians [12]. Droplet-based assays provide multiple parallel analyses in a single picolitre drop. Miniaturization of such assays can enable rapid, ultrasensitive, and multiple detection of many diagnostic biomarkers. Combining nanomaterials with such miniaturized sensing elements provides a path for the development of highly sensitive and selective biosensors [16]. Fluorescence-based detection is one of the most popular analytical tools in chemical, medical and biochemical analysis [17]. This is due to the wide range of fluorescent dyes available for biosensing detection. They are highly sensitive, non-invasive, and can be easily adapted to the sensor requirements [12]. Since 1998 quantum dots (QDs) have been established as a new type of fluorophores in biological research. Since then, semiconducting QDs have become important fluorescent probes for *in-vivo* and *in-vitro* biosensing and bioimaging [18–21]. The QDs are promising candidates for detecting biomarkers in a fast, inexpensive, precise and non-invasive manner in the early stages of cancer metastasis. Using the optimal wavelength to stimulate QD nanocrystals, different cancer growth cell biomarkers were detected, increasing the efficiency of cancer treatment. Recently, QDs have been made available for in-vivo imaging along with the delivery of therapeutic treatment to tumors. High resistance to photobleaching and high brightness make QDs suitable for visualizing single cancer cell. Also, QDs are a good candidate for multiplexed-quantitative detection of tumor biomarkers, as there is availability of better conjugation chemistry for attaching bioligands to QD’s surface [22–24].

Due to the large surface-to-volume ratio, the properties of QDs can be altered by different molecules and ions, making them sensitive to their surrounding environment. They can be attached to proteins, antibodies, and other biological molecules [25]. The well-developed surface click chemistry on the hard core-shell structure of QDs makes them attractive for preparing many types of bioconjugates like QDs conjugated with streptavidin [26]. For *in-vitro* bioanalysis, toxicity of QDs is of no concern. Hence their unique optical properties are exploited for significantly enhancing the sensitivity of bio-diagnostic and chemical assays with real-time application [27,28]. Functionalized QDs are used as labels in many biorecognition events. Antibody-conjugated QDs are the basis for fluoro-immuno-assays and DNA conjugated QDs are used as signalling probes in DNA microarrays and fluorescence *in situ* hybridization (FISH) assays. They have a higher signal-to-noise ratio (SNR) and photostability than commonly used fluorophores [29]. Quenching of emission from QDs by interfering with the electron-transfer process is used for biosensing of conformation-dependent analytes [30]. Current QDs exhibit the blinking phenomenon (rapid on-and-off light emission) which is problematic for sensing or imaging low concentrations of biomolecules. It is possible to eliminate the blinking by realizing a smooth composition the gradient from core to shell [27].

**Fig. 1.**
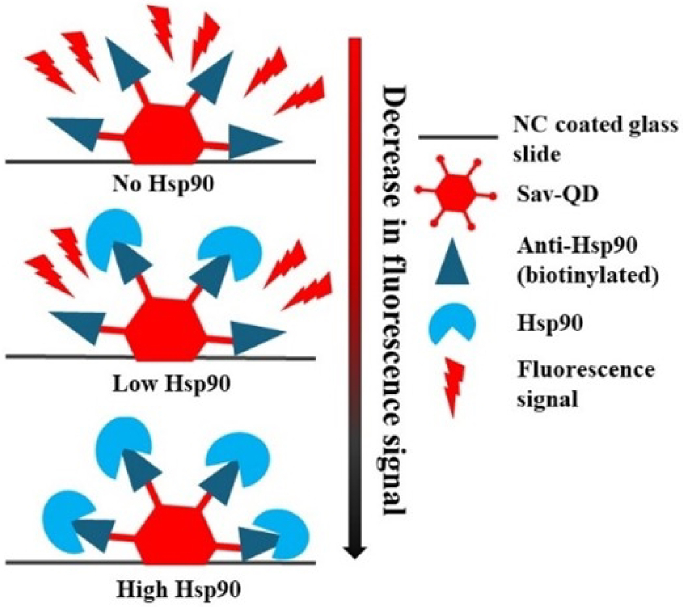
Schematic representation of the fluorescence masking of Sav-QD by different concentrations of Hsp90.

In this work, we developed a custom-built miniaturized, bench-top, and fast testing system TIQD (Transilluminated Quantum Dot) imager for reliable detection of Hsp90 in different concentrations. The measurement is based on the masking of the fluorescence of quantum dots when Hsp90 attaches to them as illustrated in Fig. 1. A CMOS sensor with cost-efficient optics and an algorithm for image processing is used for easy measurement compared to standard laboratory equipment based on photomultiplier tube (PMT) scanners. Our work is promising for developing a robust point-of-care detection system for stress and various other protein biomarkers. The development of a fast and cheap testing system for Hsp90 levels in blood plasma has thus great translational potential in stress-related diseases and even cancer detection and diagnosis in future [5].

Sav-QD solution is mixed with biotinylated antibodies for Hsp90. The reaction between streptavidin and biotin molecules attaches the antibody to the QDs. This mixture is transferred to the biochip. Hsp90 molecules bind with the antibody molecules, when the Hsp90 solution is added on the biochip. This causes fluorescence masking of the QDs, as the Hsp90 molecules prevent the photons from reaching the QD. At higher concentrations, more Hsp90 molecules are attached to the QDs, thus, the observed fluorescence masking effect is stronger.

## 2. Materials and methods

### 2.1 Measurement principles

The detection of the biomolecules in this work is based on fluorescence readout and was carried out by a developed compact TIQD imager. For comparison and reference, we used a PMT-based scanner currently being used as the standard laboratory equipment. The biochip was prepared by spotting Sav-QD and biotinylated Hsp90 antibody mixture (QD + Ab) onto the chip. The fabrication of the biochip relies on our previous work [20]. Measurements were done in two situations, before and after adding Hsp90 on the biochip, Figs. 7 and 8 show the change in the fluorescence of the QD + Ab spots before and after the Hsp90 addition. The change in the fluorescence signal (signal difference) is equivalent to the masking of the QD fluorescence induced by the Hsp90 protein binding. This is directly proportional to the concentration of Hsp90 added on the biochip. Consequently, a calculation of the limit of detection (LOD) for the TIQD imager and the PMT-based scanner is enabled.

### 2.2 Materials and setup

#### 2.2.1 Biochip fabrication materials

Sav-QD which is Streptavidin conjugated CdSe-ZnS QDs (Qdots 655) was purchased from Thermofisher (Germany), and NC glass slide (Nitrocellulose coated glass slides - 2UNY2GW00600616G) were purchased from Sartorius (Germany). Each slide has 16 nitrocellulose membrane pads (8 rows, 2 columns) with 6 mm x 6 mm dimensions. Radicicol was purchased from Biomol (Germany), γ-(6-Aminohexyl)-ATP-Cy5 was from Jena Bioscience (Germany), Biotin-Anti Hsp90 (Biotin Rabbit and Human polyclonal to Hsp90 alpha, abx445722) from Abbexa (Netherlands). The blocking buffer was the blocking solution from Candor (Germany). Human HSP90a (stock concentration 3 mg/mL or 33 µM) was recombinantly synthesized and purified in-house. Purified human blood serum (Human male AB plasma- H4522) was obtained from Sigma-Aldrich (Germany). Hitrap Blue HP affinity columns from Cytiva (via Merck, Germany) were used for filtering blood serum. PBS buffer was used for making all the mixtures and different dilutions of Hsp90. All solutions were made using doubly distilled water and analytical grade reagents.

**Fig. 2.**
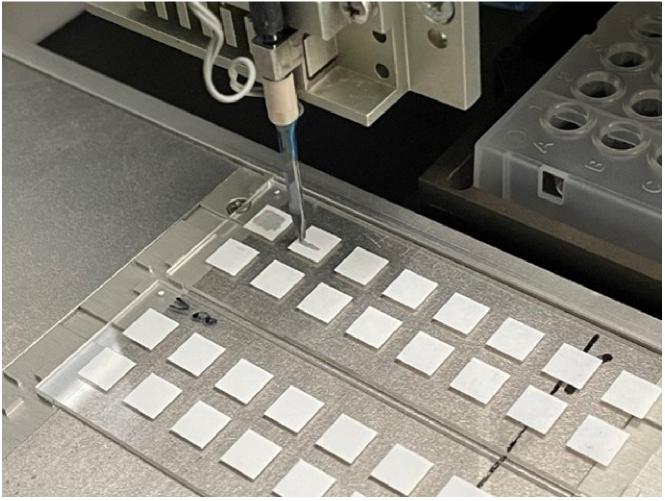
QD + Ab nM Ab mixture being printed on the nitrocellulose pads of the biochip by the pipetting tip of the piezoelectric printer (Nano-Plotter 2.1) from GeSim (Radeberg, Germany). Around 150 pL volume is spotted on each spot.

For fabrication of biochips, there are three basic printing techniques (i) micro spotting (micro-contact printing), (ii) inkjet printing and (iii) piezoelectric dispensing (non-contact). Microcontact printing has low transfer efficiency, and the transferring protocol needs to be modified for different chip substrates. Heating is involved in inkjet printing which damages the structures of heat-sensitive proteins. Piezoelectric printing uses a non-contact method that, can print small volumes down to the pL range and non-used printing samples can be recovered which renders it useful for biochip fabrication [31]. Microprinting of QD bioconjugates allows spatial capture and sensitive detection of proteins [32]. Biochip fabrication as shown in Fig. 2 was done using a piezoelectric contactless microarray printer (Nano-Plotter 2.1) from GeSim (Radeberg, Germany).

#### 2.2.2 TIQD imager

The utilized Sav-QDs exhibit a wide absorption spectrum, with a large molar absorption coefficient of approximately 1,080,000 cm^−1^M^−1^ in the ultraviolet region which decreases towards the visible wavelengths range until the emission bandwidth whose maximum is at around 655 nm [23–35]. For excitation, a diode laser source at a wavelength of 405 nm (RLDE405M-20-5, Roithner Lasertechnik GmbH, Vienna, Austria) with an output power of 20 mW is employed, ensuring a large Stokes shift. Samples containing the Sav-QD, antibodies and target molecules are trans-illuminated with the laser radiation as this configuration ensures efficient excitation, high imaging contrast and allows the source to be placed as close as possible to the test sample, see below for details. The field of view for imaging is determined as 5 mm x 5 mm based on estimating the average spot diameter of 350 µm and with a typical configuration of spots in the order of 10 rows and 1 column on each pad on the nitrocellulose coated glass slides (2UNY2GW00600616 G, Sartorius AG, Goettingen, Germany). Suitable standard optical components were used to shape the elliptical beam profile of the laser source so that uniform illumination throughout the entire field of view was achieved. A combination of a planoconvex lens (LA1951, Thorlabs GmbH, Munich, Germany), a negative meniscus lens (LF1822, Thorlabs GmbH, Munich, Germany) and a square diffuser (ED1-S50, Thorlabs GmbH, Munich, Germany) were used.

**Fig. 3.**
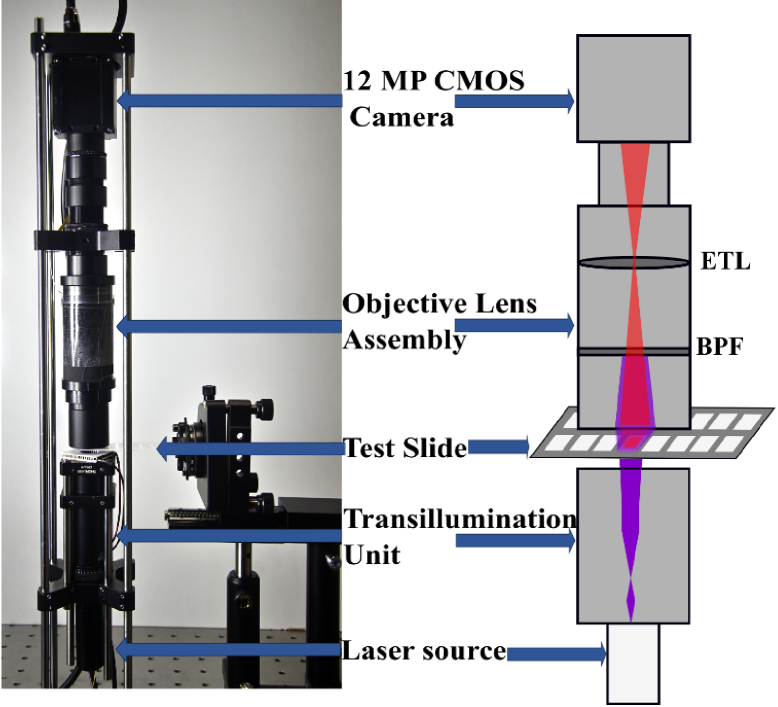
(Left) Image of the TIQD imager, testing the biochip loaded with Hsp90. (Right) Schematic layout of the imager exciting QD + Ab spots with a 405 nm laser producing 655 nm fluorescence. ETL: Electrically Tuneable Lens used for autofocussing of the test biochip, BPF: Band Pass Filter blocking the excitation light.

To realize a low cost, compact and yet reliable optical device for readout of the test samples, a commercial 1.1"inch IMX304 CMOS sensor gray-scale camera (U3-3200SE-M-GL, IDS Imaging Development Systems GmbH, Germany) with 4096 × 3000 pixels having a pixel dimension of 3.45 µm x 3.45 µm was utilized for imaging. The distance between the imaging lens and the target pads was minimized in order to achieve a higher collection efficiency of the quantum dot fluorescence. By introducing a positive meniscus lens (LE1234, Thorlabs GmbH, Munich, Germany) in the objective assembly developed for imaging, not only the spherical aberration was minimized, but also the working distance was reduced to approximately 10 mm, as verified by ray-optical simulations using the Zemax software. This led to an estimated numerical aperture (NA) of approximately 0.7, following the design criteria detailed in [36–38]. The optical setup developed also incorporates an electrically tunable lens (EL-12-30-TC-VIS-16D, Optotune, Switzerland), for automated focusing during the measurements using the focus stacking method. The light from the laser source was blocked from propagating to the camera with a narrow band-pass filter (FBH650-10, Thorlabs GmbH, Munich Germany). The final setup of the TIQD imager is as shown in Fig. 3.

### 2.3 Hsp90 purification and activity testing

Human Hsp90a (stock concentration 3 mg/mL or 33 µM) was recombinantly synthesized in *E. coli* BL21DE3 cells and purified as described earlier [38]. As a presence and activity test a microarray-based Cy5-ATP binding and Radicicol displacement assay was performed to determine the Hsp90 activity as described recently [39,40].

### 2.4 Determination of the optimal concentration of QD + Ab mixture

Different concentrations of QD + Ab mixtures, i.e., 100 nM QD + 1000 nM Ab, 75 nM QD + 750 nM Ab and 50 nM QD + 500 nM Ab, were printed on the NC glass slides. Printing was done using the above-mentioned nano-plotter and the parameters were set, so that each QD + Ab spot has a diameter around 320-350 µm. After printing biochips were fixed in an incubation chamber, so that each pad was isolated from the other. To each pad 100 µL of the blocking buffer was added. The chamber was covered with aluminium foil. Incubation was carried out at 4°C, for one hour in dark conditions. The blocking buffer was discarded, and the biochip was air dried after removing it from the incubation chamber. The biochips were initially scanned by the PMT-based scanner as a reference and then by the TIQD imager with 150 msec exposure time. This was done to check which concentration of QD + Ab mixture is suitable for both fabrication and imaging.

### 2.5 Biochip fabrication

To realize 50 µL of QD + Ab mixture, 50 nM of Sav-QD was mixed with 500 nM of Biotin-Anti HSP90 in PBS buffer. The mixture was incubated at 4 °C, with slow shaking and dark conditions for one hour. The incubation was required to enable the streptavidin-biotin interaction. Thus, the antibody was attached to QD. QD + Ab was printed on the NC slide with the microarray printer as shown in Fig. 2. The software of the microarray printer was used for setting the printing parameters such as the number of drops per spot, the number of spots and the layout of the spots on each pad. Usually, 10 spots were printed on each pad. Each spot contains 10 drops of QD + Ab mixture. The shape and size of the drop were checked by a stroboscopic camera. The total volume of each spot was approximately 150 pL and the diameter around 320 µm. Before the start of the printing process, the printing needle was washed thoroughly to remove any previous biomaterial that could affect the printing process. It was made sure that no air bubbles were trapped in the printing needle, or the tubes attached to it. After printing, the biochip was air dried so that the QD + Ab was fixed strongly on the nitrocellulose. Blocking was done to avoid unwanted binding of the target protein on the nitrocellulose membrane. For blocking, the biochip was inserted in the incubation chamber followed by incubation with the blocking buffer at 4°C for one hour in dark conditions. The buffer was discarded, and the biochip was air dried for around 15 mins. The biochip was ready for quantifying the QD fluorescence masking by different concentrations of Hsp90, bound to the Ab which are attached to the QD.

### 2.6 Systematic test of fluorescence masking depending on Hsp90 concentration

Different concentrations of Hsp90 alpha (500, 250, 100, 50, 25, 10, and 5 nM, respectively) were prepared by serial dilutions in 1X PBS buffer. These Hsp90 solutions were added to the already fabricated biochip. Each pad had a different Hsp90 concentration, and the control pad was only with PBS buffer (0 nM Hsp90). Hsp90 was incubated on the chip inside the incubation chamber, at 4°C for one hour and in dark conditions. Afterwards, Hsp90 was discarded, and the biochip was air dried for 15 mins. PMT scanning and TIQD imaging were carried out later.

### 2.7 Scanning of the biochip by PMT-based scanner

A reference scan was done by the PMT-based scanner to verify the working principle of the biochip. For excitation, a laser at 635 nm laser (33% power level corresponding to about 15 mW) was used, 350 PMT gain was set. The first scan was done before the incubation of Hsp90 (without Hsp90), and the second scan after incubation with Hsp90. The average fluorescence of all ten QD + Ab spots per pad without and with Hsp90 was calculated. Subsequently, the average signal difference for each pad was calculated by subtracting the average fluorescence values with Hsp90 from the ones without. Signal difference values were plotted against the different Hsp90 concentrations in a logarithmic scale to obtain the dose-response curves. These show the fluorescence masking effect as a function of the concentration of Hsp90. Also, this determines the detection range for the PMT-based scanner and the TIQD imager.

### 2.8 TIQD imager data processing

While the buffer solution was spotted on the nitrocellulose pads, the spots show the coffee ring effect. Deegan et al. [41] has qualitatively analysed the transport phenomenon involved in the drying of the droplet. If the area inside the droplets with pronounced coffee ring effect did not contain significant amounts of Hsp90, it was considered as background during image processing. The region in the contact line where a higher concentration of quantum dot particles was observed was considered as foreground [42]. Out of 16 pads in each of the slides, multiple images of each pad were captured by using automatic focus stacking through varying the focus of the electrically tuneable lens. The images were recorded with an exposure time of 150 msec, analogue gain of 9.02 and a digital gain of 1. The test slides were axially moved to image all pads by using a motorized stage and controller (KMTS50E/M, Thorlabs GmbH, Munich, Germany) through a USB protocol.

For each pad, seven images were captured at different focal lengths of the ETL. Area estimated gridding was executed on each image considering the number of spots on each individual pad. With each grid identified, the pixel areas were segmented as background and foreground pixels. The pixel intensity from QD was calculated as the difference between foreground and background intensity. The overall measurement taken for each pad was averaged. Subsequently, the signals were calculated for each pad with (F) and without Hsp90 (F_0_), the latter case representing the reference measurement. Dose response curves of the TIQD imager and PMT-based scanner were plotted and compared for detection range.

### 2.9 Testing Hsp90 in purified blood serum

Different concentrations of Hsp90 (1000, 750, 500, 250, 100, 75, 50, 25, 10, 7.5 and 5 nM, respectively) were made in PBS buffer and mixed with purified human blood serum. Before mixing, the blood serum was filtered out to remove unwanted albumin proteins that might cause background noise. This mixture was added to the biochip and incubated for one hour at 4 °C and dark conditions. Afterwards, the results from the biochip were analyzed to quantify the fluorescence masking and determine the limit of detection for PMT-based scanner and TIQD imager, respectively. Dose-response curves and Stern-Volmer plots were created. For each Hsp90 concentration value, results from two chips were averaged. The chips were subject to the same conditions. The sensitivities of both systems are calculated.

## 3. Results

### 3.1 Testing Hsp90 activity

**Fig. 4.**
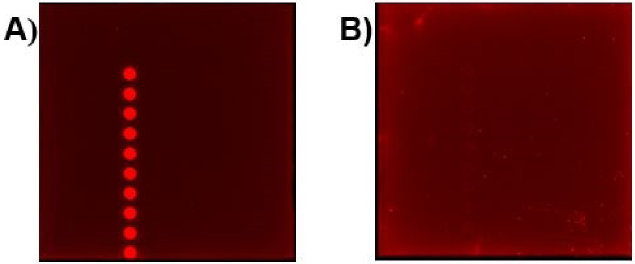
Testing the activity of the in-house produced Hsp90 [20]. a) Spotted Hsp90 on the NC slide showing binding with 100 nM Cy5-ATP. b) In the presence of 1 µM Radicicol, there is incomplete inhibition of binding of Cy5-ATP.

As seen in Fig. 4, Hsp90 showed binding with 100 nM Cy5 labelled ATP. There was complete inhibition of ATP binding in the presence of 1 µM Radicicol. This proves that the in-house purified Hsp90 is active.

### 3.2 Determining suitable QD + Ab concentration for TIQD imager

**Fig. 5.**
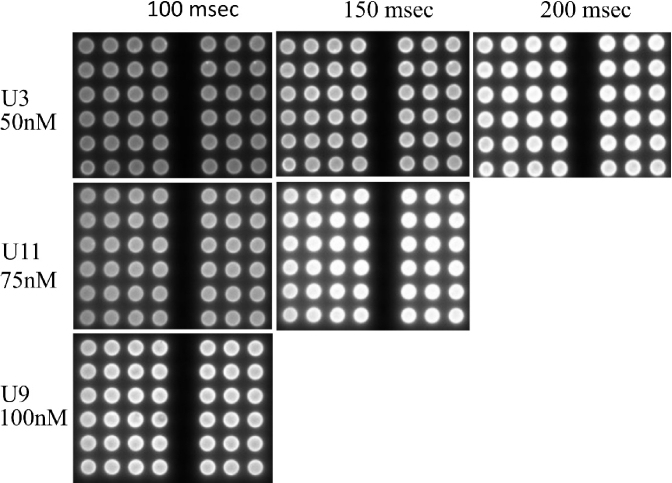
Biochip U3 with 50 nM QD + 500 nM Ab, U11 with 75 nM QD + 750 nM Ab and U9 100 nM QD + 1000 nM Ab spots. TIQD images taken at 100 msec and 150 msec exposure time. 50 nM QD + 500 nM Ab mixture is selected for fabricating the biochip and 150 msec exposure time selected for TIQD imaging.

Printing of the 100 nM QD + 1000 nM Ab mixture was difficult compared to the case of 75 nM QD + 750 nM Ab and 50 nM QD + 500 nM Ab. The higher concentrated mixture was blocking the nano plotter pipette tip. After imaging with the TIQD imager, as shown in Fig. 5, for different QD and Ab concentrations and with 100 msec and 150 msec exposure time, respectively, the background fluorescence obtained was minimal for images captured with an exposure time of 150 msec and a concentration of 50 nM for the QDs. Hence, the 50 nM QD + 500 nM Ab mixture was selected for the further work.

### 3.3 Measurement of fluorescence masking using standard PMT-based scanner and TIQD imager

**Fig. 6.**
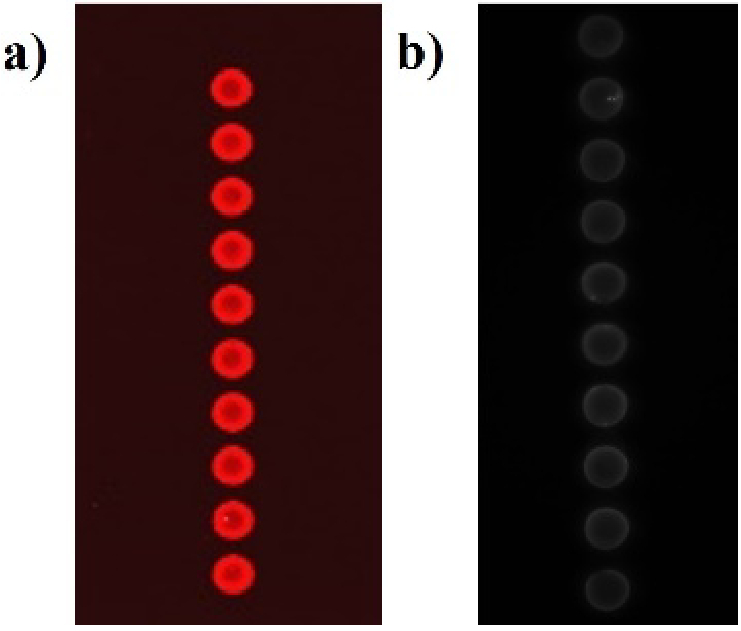
Image of one of the pads with 10 QD + Ab spots taken a) by the PMT-based scanner and b) the TIQD imager.

**Fig. 7.**
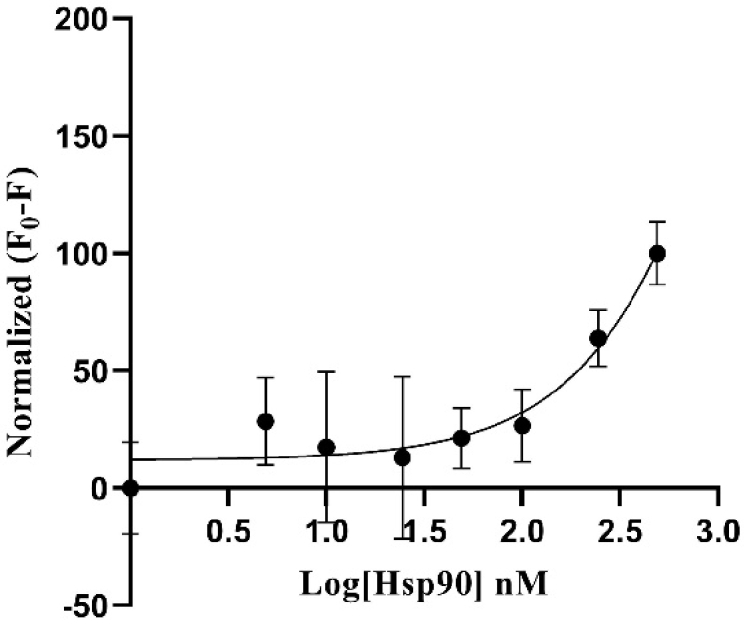
Fluorescence signal difference (between F_0 _= signal without Hsp90 and F = signal with Hsp90, in PBS) was normalized plotted against different concentrations of Hsp90 in a dose-response curve. Fluorescence signal was recorded with the PMT-based scanner. The higher the concentration of Hsp90, the larger is the signal difference, hence the higher the masking of the fluorescence of QD (50 nM) + Ab (500 nM) spots. Maximum signal difference is counted as 100% for 500 nM Hsp90 and 0% for 0 nM Hsp90. Detection range for the PMT-based scanner lies between 1000 to 50 nM Hsp90. The black curve is a three parameter dose-response function fitted to the data points.

**Fig. 8.**
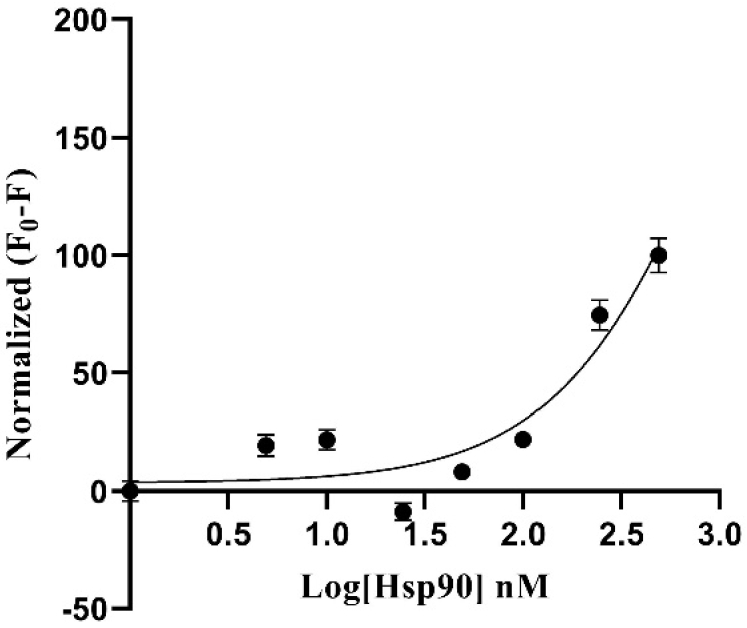
Fluorescence signal difference (F_0 _= signal without Hsp90 and F = signal with Hsp90, in PBS) was normalized plotted against different concentrations of Hsp90 in a dose-response curve. Fluorescence signal was recorded with the TIQD imager. The higher the concentration of Hsp90, the larger is the signal difference, hence the higher the masking of the fluorescence of QD (50 nM) + Ab (500 nM) spots. Maximum signal difference is counted as 100% for 500 nM Hsp90 and 0% for 0 nM Hsp90. Detection range for the TIQD imager lies between 1000 to 50 nM Hsp90. The black curve is a three parameter dose-response function fitted to the data points. A few data points have very small standard deviations, hence the bars are not visible.

As can be seen in Fig. 6, the image obtained from the PMT-based scanner exhibits more fluorescence intensity compared to the TIQD images. As expected in both cases, the signal difference values were directly proportional to the Hsp90 concentration, i.e., a decrease in fluorescence was observed with decrease in Hsp90 concentration from 500 nM to 0 nM, see the dose-response curves in Figs. 7 and 8. The detection range for both PMT-based scanner and TIQD imager lies between 1000 to 50 nM of pure Hsp90 in PBS buffer. The signal from the TIQD measurement at a concentration of 25 nM Hsp90 is lower than the reference value due to readout noise and printing inaccuracies leading to unwanted bright or dark regions in the QD + Ab spots and thus more noisy results. For the same reasons, the measurement uncertainties for the PMT scanner are larger at some Hsp90 concentrations.

### 3.4 Measurement of fluorescence masking for Hsp90 mixed in human blood serum

**Fig. 9.**
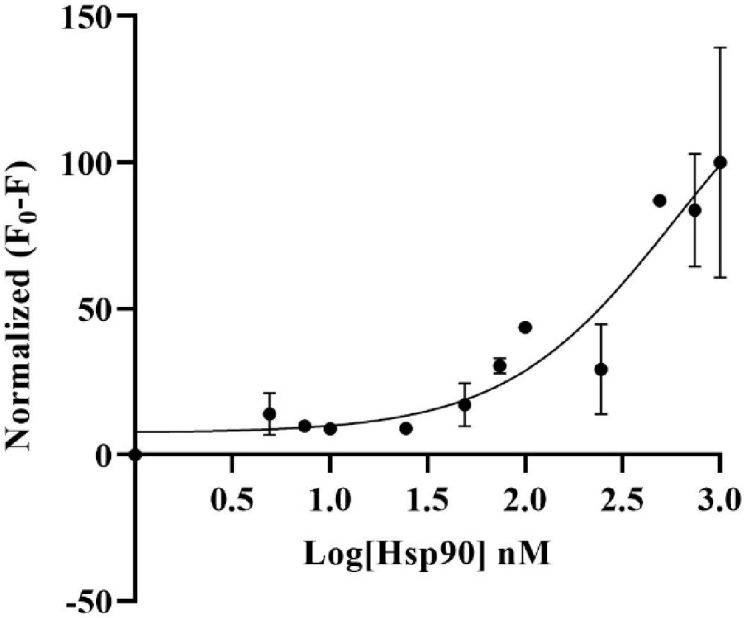
Fluorescence signal difference (F_0 _= signal without Hsp90 and F = signal with Hsp90) was normalized plotted against different concentrations of Hsp90 mixed with human blood serum in a dose-response curve. Fluorescence signal was recorded with the PMT-based scanner. The higher the concentration of Hsp90, the larger is the signal difference, hence the higher the masking of the fluorescence of QD (50 nM) + Ab (500 nM) spots. Maximum signal difference is counted as 100% for 1000 nM Hsp90 and 0% for 0 nM Hsp90. Detection range for the PMT-based scanner lies between 1000 to 50 nM Hsp90. The black curve is a three parameter dose-response function fitted to the data points. A few data points have very small standard deviations, hence the bars are not visible.

**Fig. 10.**
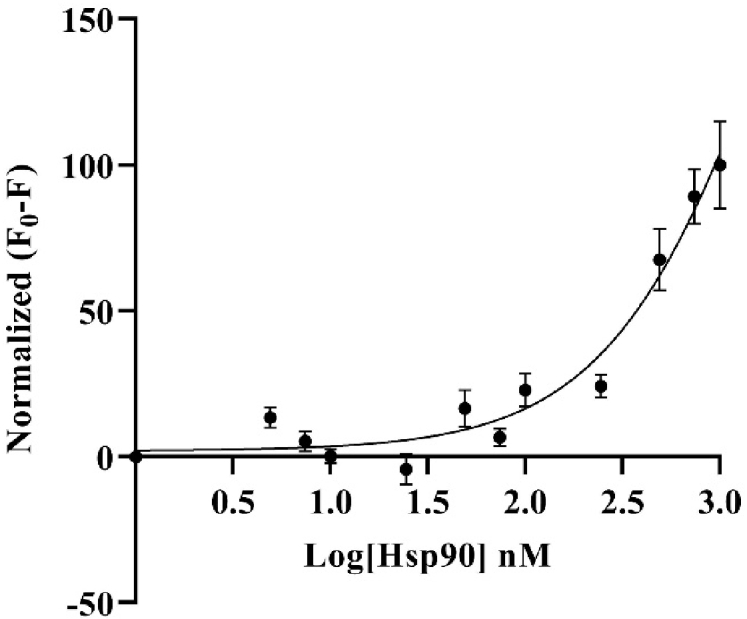
Fluorescence signal difference (F_0 _= signal without Hsp90 and F = signal with Hsp90) was normalized plotted against different concentrations of Hsp90 mixed with human blood serum in a dose-response curve. Fluorescence signal was recorded with the TIQD imager. The higher the concentration of Hsp90, the larger is the signal difference, hence the higher the masking of the fluorescence of QD (50 nM) + Ab (500 nM) spots. Maximum signal difference is counted as 100% for 1000 nM Hsp90 and 0% for 0 nM Hsp90. The detection range for the TIQD imager lies between 1000 to 50 nM Hsp90. The black curve is a three parameter dose-response function fitted to the data points.

As seen in Figs. 9 and 10, Hsp90 in blood serum exhibited similar fluorescence masking as Hsp90 in PBS buffer. The detection range for both the PMT-based scanner and TIQD imager was between 1000 nM to 50 nM in this case. The overall masking effect of blood serum was slightly higher than that of PBS buffer. This is due to the presence of bigger biomolecules or proteins in the blood serum. For each data point in the graphs shown below, an average of measurements from different biochips was taken, the latter being processed in the same way. The standard deviation values are higher for the PMT scanner due to above mentioned readout noise and inaccuracies of the microarray printer.

### 3.5 Limit-of-detection of PMT-based scanner and TIQD imager

**Fig. 11.**
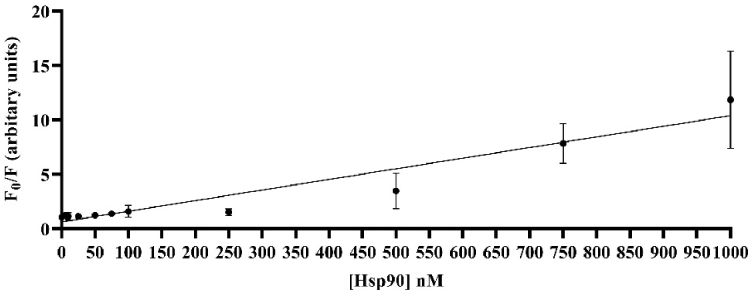
Linear regression (black line) for the PMT-based scanner data. (F_0_/F) plotted versus Hsp90 concentration ranging from 1000 to 0 nM. F_0_ and F are fluorescence intensities of QD + Ab spots with and without Hsp90. The inverse of the slope is k^-1 ^= 102.4 nM^-1^ and r^2 ^= 0.9251. LOD of PMT-based scanner is around 37.47 nM. Few data points have very small standard deviations, hence the bars are not visible.

**Fig. 12.**
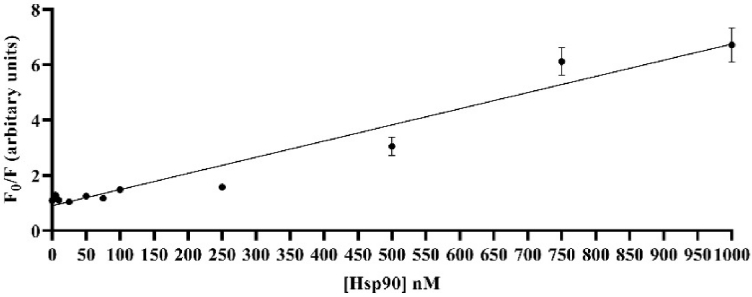
Linear regression (black line) for the TIQD imager data. (F_0_/F) plotted versus Hsp90 concentration ranging from 1000 to 0 nM. F_0_ and F are fluorescence intensities of QD + Ab spots with and without Hsp90. The inverse of the slope is k^-1^ = 171 nM^-1^ and r^2^ = 0.9518. LOD of TIQD imager is around 11.8 nM. Few data points have very small standard deviations, hence the bars are not visible.

From the SV plots of the PMT-based scanner in Fig. 11 and the TIQD imager in Fig. 12 and by using Eq. (1), we calculated the LODs for both cases according to:

(1) $$LOD=\frac{3\times \mathrm{standard deviation of control}\;(0\;\text{nM}\;\text{Hsp}90)\;}{\mathrm{slope of SV linear regression}\;[\text{n}{\text{M}}^{-1}]}$$

**Fig. 13.**
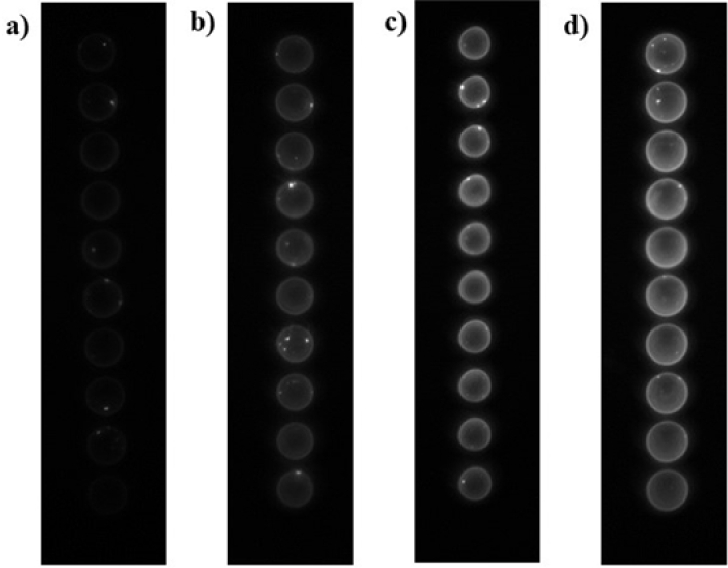
Images taken by the TIQD imager after different Hsp90 concentrations were added to the blood serum. a) 1000 nM, b) 250 nM, c) 50 nM, d) only blood serum (0 nM).

We found LODs of 37.47 nM and 11.8 nM for the PMT-based scanner and the TIQD imager, respectively. The CMOS camera of the TIQD system is not as sensitive as the PMT-based scanner for darker images, see Fig. 13 as example. In general, the data analysis for the PMT and TIQD measurements at higher concentrations of Hsp90 (approximately 1000 nM to 500 nM) can be optimized in future, e.g., by leaving out darker areas in the spots. Overall, the LOD values of both systems are comparable. The TIQD system appears even more feasible for lower concentrations which is beneficial for in-field application cases. The sensitivity *S* of both systems were calculated according to:

(2) $$S=\frac{\mathrm{slope of SV linear regression}\;[\text{n}{\text{M}}^{-1}]}{\mathrm{active surface spot area}\;A\;[\mu {\text{m}}^{2}]}$$

The active area *A* of each QD + Ab spot with a diameter of approximately 320 µm is around 80,000 µm^2^. For 10 spots per pad, the total active area amounts to around 800,000 µm^2^. Using Eq. (2), we found a sensitivity *S* of 0.0078 nM^-1^mm^-2^ for the PMT-based scanner system. The TIQD imager is slightly more sensitive, with a sensitivity S of 0.00047 nM^-1^mm^-2^.

## 4. Discussion

The developed simple and compact optical TIQD imager for sensitive detection of Hsp90 concentrations on a biochip worked well in the studied range of concentrations. This was verified through a comparison of the measurements with those from a commercial and expensive PMT-based scanner. In combination with our sensor strategy by masking the QD fluorescence we have developed a new tool suitable for future multiplex testing of biomarkers. For this, the signal differences between samples with and without Hsp90 were observed and the fluorescence masking of printed 50 nM QD + 500 nM Ab on NC coated slides was determined. Using a higher concentration of QD + Ab mixture (e.g., 100 nM/ 75 nM QD + 1000 nM/750 nM Ab) the signal difference can be measured even at higher concentrations of Hsp90. However, the printing of the mixture at higher concentrations was difficult due to particle aggregation in the printing needle. Also, the signal from these mixtures on the biochip was very high when analyzed by the PMT-based scanner which made a comparison with the TIQD imager data difficult. A combination of 50 nM QD + 500 nM Ab was best suitable for printing, scanning, and imaging in the current setup configuration. Also, with an exposure time of 250 msec, we obtained minimal background fluorescence and the image segmentation for extracting average spot intensities was improved.

For the PMT-based scanner and the TIQD imager, the lower detection range is around 50 nM of Hsp90 in blood serum for 50 nM QD concentration. This is due to high fluorescence signal levels making it difficult to detect the masking effect at lower concentrations of Hsp90, as seen in Figs. 7 and 8. Random noise from the printing of the chip, buffer and Hsp90 were also observed. Buffer conditions and QD concentrations need to be optimized to facilitate better comparison between the PMT and TIQD data. Overall, the TIQD imager can detect slightly lower concentrations of Hsp90 compared to the PMT-based scanner. This can be due to the different fluorescence intensities of the two systems. The PMT-based scanner is more sensitive at low light conditions as it has high gain and low noise in comparison to the CMOS sensor of the TIQD imager. Further investigations on these dependencies will be performed in the next steps. Overall, the LOD of the TIQD was lower and its sensitivity *S* was higher compared to the PMT-based system, thus making it more suitable for the detection of lower Hsp90 concentrations.

The future work will focus on the optimization of the biochip fabrication to detect multiple biomarkers in biological samples at even lower concentrations. The optical setup will be further miniaturized, and the opportunities for additive manufacturing will be exploited for this purpose. A miniaturized version of the system might be read out by a smartphone camera so that the costs for the system could be reduced [21,43]. We will also aim to lower the limit of detection. Furthermore, the QD concentration in the spots can be optimized, by adding an additional layer of antibodies or proteins such as Thyroid hormone receptor beta binding to Hsp90 [44]. Also, the background noise and coffee ring effect might be reduced by using different substrates for the biochip.

## 5. Conclusion

In conclusion, we developed a low-cost TIQD imager that was able to detect the masking of Sav-QD fluorescence as a function of different concentrations of Hsp90. The detection measurement range of the TIQD imager was comparable with that of the standard PMT-based scanner, their limit of the detection was found to be nearby values. The TIQD imager can be used for detection reliably for lower concentrations of Hsp90. Non-contact printing was used for the fabrication of the quantum dot-based sensor. Sav-QD with biotinylated Hsp90 antibody was printed on the biochip and different concentrations of Hsp90 were added. Hsp90 attached to the printed QD + Ab via antigen-antibody interaction led to fluorescence masking as obvious from the signal difference without and with Hsp90. A direct proportionality with Hsp90 concentration was found. With additional optimization of the biochip and the TIQD imager and with advanced data analysis, Hsp90 and similar stress biomarkers could be detected in biological samples at low concentrations, potentially down to the pM regime. A point-of-care quantum dot-based rapid test appears possible with long shelf lifetimes and multiple usage of the same biochip by a single user. For this purpose, the integration into a hand-held system, e.g., a mobile phone device, appears to be beneficial.

## Data Availability

Data underlying the results presented in this paper can be obtained from the authors upon reasonable request.
